# The embryology, metamorphosis, and muscle development of *Schizocardium karankawa* sp. nov. (Enteropneusta) from the Gulf of Mexico

**DOI:** 10.1186/s13227-023-00212-0

**Published:** 2023-04-19

**Authors:** Noura Jabr, Paul Gonzalez, Kevin M. Kocot, Christopher B. Cameron

**Affiliations:** 1 Département de Sciences Biologiques, Université de Montréal, C.P. 6128, Succ. Centre-Ville, Montréal, QC H3C 3J7 Canada; 2 Computational and Statistical Genomics Branch, Division of Intramural Research, National Human Genome Research Institute, National Institutes of Health, Bethesda, MD 20892 USA; 3 Department of Biological Sciences and Alabama Museum of Natural History, University of Alabama, Tuscaloosa, Alabama 35487 USA

**Keywords:** Enteropneusta, Acorn worm, Spengelidae

## Abstract

*Schizocardium karankawa* sp. nov. has been collected from subtidal muds of the Laguna Madre, Texas, and the Mississippi coast, Gulf of Mexico. The Texas population is reproductive from early February to mid-April. Gametes are liberated by a small incision in a gonad. Oocyte germinal vesicle breakdown is increased in the presence of sperm, and the highest fertilization success was in the artificial seawater Jamarin U. Manually dechorionated embryos develop normally. Development was asynchronous via a tornaria larva, metamorphosis and maintained to the juvenile worm 6 gill-pore stage. Phalloidin-labeled late-stage tornaria revealed retractor muscles that connect the pericardial sac with the apical tuft anteriorly, the oesophagus ventrally, and muscle cells of the early mesocoels. The muscle development of early juvenile worms began with dorso-lateral trunk muscles, lateral trunk bands, and sphincters around the gill pores and anus. Adult worms are characterized by a stomochord that bifurcates anteriorly into paired vermiform processes, gill bars that extend almost the entire dorsal to ventral branchial region resulting in a narrow ventral hypobranchial ridge, and an elaborate epibranchial organ with six zones of discrete cell types. The trunk has up to three rows of liver sacs, and lateral gonads. The acorn worm evo-devo model species *Saccoglossus kowalevskii*, *Ptychodera flava*, and *Schizocardium* *californicum* are phylogenetically distant with disparate life histories. *S. karnakawa* from *S.* *californicum* are phylogenetically close, and differences between them that become apparent as adult worms include the number of gill pores and hepatic sacs, and elaborations of the heart–kidney–stomochord complex. An important challenge for evolutionary developmental biology is to form links from phylogenetically distant and large-scale differences to phylogenetically close and small-scale differences. This description of the embryology, development, and adult morphology of *S. karankawa* permits investigations into how acorn worm development evolves at fine scales.

**Supplementary Information:**

The online version contains supplementary material available at 10.1186/s13227-023-00212-0.

## Background

Deuterostomia is comprised of the hemichordates and echinoderms, together called Ambulacraria, which is the sister group to the chordates. The body plans of these groups are highly divergent, but comparative morphology, fossils and molecular developmental studies are converging on the hypothesis of an acorn worm-like ancestor. Acorn worm embryonic development, tornaria larva, and tricoelomic body plan are comparable to those of echinoderms, whereas the adult worms, with a pharynx perforated with gill slits, are comparable to the chordate body plan. Precise details of the similarities and differences of these traits have come from a growing list of acorn worm species that have had their development described in the laboratory. These include *Saccoglossus kowalevskii* [1–3], *Harrimania planktophilus*,[4] *Schizocardium californicum* [5, 6], *Glandiceps hacksi* [7], *Ptychodera flava* [8, 9], *Balanoglossus simodensis* [10] [11], and *Balanoglossus misakiensis* [12, 13]. Other species for which something is known of their development, based on life-history stages collected from the plankton, include two unknown species of *Balanoglossus* [14, 15] and *Glandicepts stiasnyi* [16]. The development of these species has permitted direct comparisons of embryogenesis, nervous system development, and gene expression between acorn worm genera.

Some insights on deuterostome molecular evolution and development that have come from cross-phyletic comparisons with acorn worms include studies of axial patterning, endomesoderm and mesoderm specification, nervous systems patterning (including dorsal cord neurulation), pharynx and gill pore development, reviewed in [17], and regeneration, reviewed in [18]. Two acorn worm species have contributed the most to these insights, the direct developer *Saccoglossus kowalevskii* (family Harrimaniidae) and the indirect developer *Ptychodera flava* (family Ptychoderidae). *Saccoglossus kowalevskii* develops directly from relatively large eggs (~ 300 μm), through embryo to the juvenile stage without an intervening planktotrophic larval stage [19]. However, it does have a short-lived, non-feeding stage, with a ciliated telotrochal swimming band composed of multiciliated cells. By comparison, *Ptychodera flava* displays indirect development. It begins as a small, fertilized egg (~ 120 μm), that develops into a planktonic, feeding larva called a tornaria, with a telotroch, a ciliated apical sensory organ, oral and aboral ciliated feeding bands, and a complete gut. Like *Saccoglossus,* it has a posterior telotroch that turns the larva as it swims, from where tornaria gets its name. The genomes of *Saccoglossus kowalevskii* and *Ptychodera flava* are publicly available and have revealed details about the gene set of the deuterostome ancestor, conserved gene linkage in the deuterostomes, a deuterostome pharyngeal gene cluster, and several deuterostome gene novelties [20]. These two acorn worms are separated by at least 370 million years of evolution. Further comparative work with these species should inform hypotheses of larval evolution, conserved traits between acorn worms and echinoderm larvae, and how two species from the same phyla with distinct life histories develop into remarkably similar adult forms.

A third emerging evolutionary developmental model acorn worm species is a member of the family Spengelidae, *Schizocardium californicum* [5, 6]. Spengelids represent an intermediate between the families Harrimaniidae and Ptychoderidae [21, 22]. Like ptychoderids, spengelids develop via a tornaria larva, but the adults, depending on the genus, may lack buccal diverticula, gill bar synaptaculae, hepatic sacs or collar nerve roots that characterize ptychoderids [21]. The emergence of this model is important to understand if this similar developmental mode has broadly shared developmental mechanisms, representing conserved traits between the phylogenetically close families Ptychoderidae and Spengelidae [23, 24]. This two-species comparison may also be expected to lead to discoveries about the developmental genetic and cellular basis of parallelism [25]. The abundant little bridges (synapticles) that connect the primary and secondary gill bars of *Schizocardium* are not shared with other spengelids but are present in all ptychoderids, including *Ptychodera flava*. Do these morphologically analogous morphologies develop via homologous genes or components of gene regulatory networks? Morphological traits unique to *Schizocardium* are mostly known from the taxonomic literature and, therefore, from adult worms. *Schizocardium* has an anterior vermiform extension of the stomochord, a heart–kidney stomochord complex (a homologue of the echinoderm axial complex) that bifurcates anteriorly, and gill bars that extend so far ventrally into the digestive pharynx that the pharynx is reduced to a diminutive hypobranchial strip. The dorsal pharynx has an epibranchial ridge with discrete zones of cells that may be homologous to the chordate endostyle [26].

Here we provide an account of the discovery of *Schizocardium karankawa* sp. nov. and its reproductive season, conditions for fertilization, method of dechorionation, and development, emphasising muscle development from tornaria to the juvenile worm. The establishment of this new developmental model is significant, because it is amenable to experimentation (i.e., cell injections, microsurgery) and provides a direct comparison with the closely related *Schizocardium californicum* [5, 6, 27]. Our molecular phylogenetic analyses confirm the close relationship between these species, which permits investigations into whether similar molecular-developmental processes regulate acorn worm development. The expectation is that these processes will be more conserved between two species that speciated more recently. This comparison may also reveal what genes, or alleles, defined the microevolutionary events resulting in their relatively recent speciation [28]. Here, we provide a detailed comparison from fertilization, embryogenesis, larval development, metamorphosis, juvenile development, and adult morphology. We provide new data on acorn worm muscle development and new phylogenetic trees of the acorn worm clade. This study increases the number of spengelids to 21 species and the genus *Schizocardium* to four species, with names that reflect their collection locations.

## Materials and methods

*Schizocardium karankawa* were collected in Corpus Christi Bay, Texas using a Peterson grab at green marker channel 71 (2.2 worms/grab) and red marker channel 66B (0.8 worms/grab), locations previously reported in [29, 30], between on February 7 and April 10, 2001. The worms used in this study, including the holotype, were collected from fine mud. It is likely common throughout the subtidal Gulf of Mexico. Embryos were reared at the University of Texas Marine Science Institute (UTMSI). They were used to document the development from embryos to tornaria. More animals were collected from March 13 to April 12, 2013, and these animals were used for taxonomy and to document development from mid-tornaria through to juvenile worms. Eggs were fertilized at room temperature in desk-top bowls. The bowls were then put in a shallow water flow through sea-table for subsequent developmental stages. The natural temperature of the cultures were cooler for the February 2001 season compared to the March 2013 season.

## Scanning electron microscopy (SEM) and histological sections

Larvae and juveniles were anaesthetized, fixed in Millonig’s buffered 2.5% glutaraldehyde, and post-fixed in bicarbonate-buffered 2% osmium tetroxide. Specimens were observed with a Hitachi S-3500N SEM in the biology department at the University of Victoria following dehydration in ethanol, critical point drying from liquid carbon dioxide, transferred individually to double-sided tape on an SEM stub, and sputter coated with gold.

For histology, adult specimens were dehydrated, paraffin-embedded, trimmed, and sectioned at 10–12 μm, mounted onto slides, dewaxed, and rehydrated, trichrome stained, and photographed on a compound or dissecting microscope. The staining steps included placing slides for 15 min in Bouin’s solution at 56 °C, followed by Weigert’s Iron Hematoxylin for 5 min, then Biebrich scarlet–acid fuchsin for 5 min, phosphotungstic–phosphomolybdic acid solution for 5 min, and finally to aniline blue solution for 5 min. The sectioned and stained specimens were viewed with an Olympus SZX16 stereomicroscope or an Olympus BX51 compound microscope. Select sections were photographed with a Q Imaging Retiga-2000R digital camera using Q Capture Pro software by Q Imaging following [31].

## Labeling with phalloidin and confocal microscopy

Embryos and juvenile worms were fixed in MOPS-buffered 2.5% paraformaldehyde, rinsed, and stored in phosphate buffered saline (PBS, pH 7.4) containing 0.1% sodium azide for several days to 1.5 months at 6–8 °C before further processing. All steps in the labeling procedure were done at 6–8 °C on an orbital shaker. Specimens were rinsed and labeled in phalloidin solution for 20 min, rinsed and then images of labeled specimens were obtained with a Zeiss LSM410 confocal scanning laser microscope. Maximum projection reconstructions were generated from multiple focal planes at intervals ranging from 1 to 3 mm.

## Molecular phylogeny

### Phylogenetic analysis of 16S

All publicly available mitochondrial large subunit ribosomal RNA gene (16S) sequences from representatives of Spengelidae (*Glandiceps abyssicola*—KC776732.1; *Glandiceps hacksi*—JN886755.1; *Schizocardium* cf. *brasiliense*—MH841936.1; *Schizocardium karanakawa*—extracted from PRJNA892992; *Schizocardium californicum*—extracted from SRR2922012) plus two representatives of Ptychoderidae (*Ptychodera flava*—LC018637.1; *Glossobalanus marginatus*—MG652439.1) were downloaded from NCBI. These were used as query sequences to extract a full-length 16S sequence from the *Schizocardium karankawa* transcriptome using BLASTN [32]. Sequences were aligned in MAFFT 7.310 [33] using the “auto” setting. Uncorrected pairwise distances between *Schizocardium karankawa* and other *Schizocardium* 16S sequences were calculated using MEGA 7.0.26 [34]. Phylogenetic analysis of 16S sequences was conducted using maximum likelihood in IQ-Tree 2 [35] with the best-fitting model for each partition (-m MFP). Topological support was assessed with 1,000 rapid bootstraps. Ptychoderidae was used to root the tree.

### Phylogenomic analyses

Our orthology inference and matrix construction approach followed the approach of [36]. Publicly available hemichordate transcriptomes were downloaded from NCBI SRA as raw reads except for the assembled transcriptome of *Ptychodera flava*, which was downloaded from NCBI TSA. For *Sacoglossus kowalevskii* and the echinoderm outgroups, protein sequences were downloaded from NCBI Genome (see Table 1). Transcriptomes were assembled de novo using Trinity 2.10 [37] with the default settings for quality/adapter trimming and digital normalization, except that a file containing the SMART-Seq adapter sequence used by [22] was provided for adapter trimming. Transcriptome assemblies were then translated using the TransDecoder version bundled with Trinity 2.10.

**Table 1:** Timetable of developmental stages of *Schizocardium karanakawa* at 19 °C and 23 °C

Developmental stage	February 25 at 19 °C	April 10 at 23 °C
1st polar body stage embryos are slightly negatively buoyant	50 min	
2nd polar body	1 h 10 min	
2-cell	2 h	1 h 30 min
4-cell	2 h 45 min	
8-cell	3 h 20 min	2 h
16-cell	3 h 55 min	2 h 25 min
32-cell stage embryos are neutral to positively buoyant		
Early blastula	8 h	4 h 15 min
Late blastula	10 h	5 h 20 min
Mid-gastrula (hatching)	17 h	
Late gastrula (ballooning)	20 h	
Proboscis pore-stage (Fig. 3I)	22 h 30 min	9 h

Protein sequences from the newly generated *Schizocardium karankawa* transcriptome were combined with amino acid sequences from the 22 other hemichordate and echinoderm (outgroup) taxa. We used OrthoFinder 2.4.0 [38] to identify groups of homologous sequences (HomoGroups) among taxa. We then processed the resulting HomoGroups to exclude sequences and entire alignments unsuitable for phylogenetic analysis. First, individual sequences < 100 amino acids and HomoGroups sampled for fewer than four taxa were excluded from the analysis. Remaining HomoGroups were then aligned with MAFFT 7.310 [33], putatively mistranslated regions were removed with HmmCleaner [39], and alignments were trimmed to remove ambiguously aligned regions with BMGE 1.12.2 [40]. Approximately ML trees were then constructed for each alignment with FastTree 2 [41] with the –slow and –gamma options, and PhyloPyPruner 0.9.5 (https://pypi.org/project/phylopypruner) was used to identify sets of strictly orthologous sequences (OrthoGroups) with the following settings: –min-taxa 4, –min-support 0.9, –mask pdist, –trim-lb 3, –trim-divergent 0.75, –min-pdist 0.01, –prune LS.

We performed a maximum likelihood analysis on the resulting phylogenomic data set in IQ-Tree 2 [35] on the University of Alabama High-Performance Computing (UAHPC) cluster. The matrix was partitioned by gene with unlinked partitions (-Q) using the best-fitting model of amino acid substitution for each partition (-m MFP). Topological support was assessed with 1,000 rapid bootstraps. Echinodermata was used to root the tree.

1) Systematic diagnoses of the Spengelidae and its genera.

**Class Enteropneusta Gegenbaur, 1870** [42]

**Family Spengelidae Willey 1899** [43]

(Glandicipitidae Spengel 1901) [44]

**Diagnosis** The family is characterized by the occurrence in all its members, of an anterior vermiform process of the stomochord and a circular muscle fibre layer inside the longitudinal muscle fibre layer in the trunk. The skeletal cornua usually extend over the whole length of collar. Dorsal nerve roots arising from the collar nerve cord are rare. The lateral septum is absent. Hepatic caeca and synapticula may or may not be present. In those cases where the development has been studied, the life history has a typical tornaria larva [21, 43, 45].

**Remarks** Of the four known genera included in this family—*Schizocardium*, *Willeyia*, *Glandiceps* and *Spengelia*, only one species, a result of the present work, *Schizocardium karnakawa* is known from the Gulf of Mexico.

**Genus Schizocardium Spengel, 1893**

**Type species. Schizocardium brasiliense (Spengel, 1893)**

**Diagnosis** The stomochord bifurcates anteriorly and extends into the proboscis coelom as paired lateral diverticula. The cardiac vesicle extends anteriorly as a pair of long tubes and is surrounded by a paired glomerulus. The ventral septum of the proboscis extends to the tip of this process. Peribuccal cavities extend forward from the trunk coelom. The collar is broader than long. The genus is distinguished by the fact that the gills are large and comprise virtually the whole wall of the pharynx in transverse section. Because of this, the midventral digestive pharynx is only a narrow hypobranchial strip. There is a well-developed mushroom-shaped dorsal epibranchial ridge that project into the pharynx lumen that has several distinct zones of cells (see [26]). Peripharyngeal cavities are present in the pharynx tongue bars. Collagenous synapticula provide a bridge between primary and secondary (tongue) gill bars. Only lateral gonads occur. The lateral genital ridges may be conspicuous posterior to the branchial region. The hepatic caeca are present as two discrete rows of sacs. Anterior and posterior intestinal pores are present.

**Remarks** The genus now includes the following species: *S. peruvianum* (Spengel, 1893) [46], *S. brasiliense* (Spengel, 1893) [46], *S. californicum*, Cameron & Perez, 2012 [21] *Schizocardium karankawa.* sp. nov., Cameron.

**Etymology of the species name:** *S. karankawa* were first collected along the South Texas coast, home of the Karankawa First Nations people, from whom it gets its name. Its known range extends to Mississippi, where it was collected at 12.5 m depth by [22] and referred to as *S.* cf. *brasiliense*.

**Diagnosis** The primary and secondary gill bars of *S. karankawa* are connected by approximately 40 synapticula. *S. peruvianum* has 30 and *S. californicum* has 10–20. The number is not reported for *S. brasiliense*. Other *Schizocardium* species have two discrete rows of hepatic sacs, but *S. karankawa* has one, two, or three rows of sacs, depending on the position along the digestive trunk. The epibranchial ridge is long and large compared to the other species.

**Comments** The following is a list of differences between *S. karnakawa* and its sister species *S. brasiliense.*

*S. karnakawa* sp. nov*.*:

1. The proboscis ciliated epidermis was 400–500 μm thick.
2. The proboscis circular muscle layer was the same as the epidermis.
3. Limited ventral septum of proboscis.
4. No anterior ventral collar septum (mesentery).
5. The first third of the hepatic sacs were paired, then two pairs of sacs, then mid-hepatic region the sacs were arranged in rows of three to four pairs. The posterior third of the hepatic region had one or two pairs, the outer more well-developed than the inner which were small and oval.

*S. brasiliense* (Spengel, 1893, Cameron & Perez 2012)*:*

1. The proboscis ciliated epidermis was 80–100 μm thick.
2. The proboscis circular muscle layer was 200 μm and decreased to 60 μm.
3. Well development ventral proboscis septum (mesentery).
4. Dorsal and ventral septa begin at the anterior collar and are complete.
5. The hepatic caeca are present as two separate pairs of sacs.

### Material examined

Adult worms were collected by CBC (in 2001) and CBC and PG (in 2013). The holotype was relaxed in MgCl_2_, and fixed in Bouin’s solution for histology, and RNAlater for sequencing. For type locality, see Materials and Methods. The holotype consists of 281 histological slides, curated at the National Museum of Natural History (Smithsonian Institution).

#### Experimental manipulations

To explore this animal’s development and increase the efficacy of fertilization events, three small experiments were done to increase sperm motility, fertilization, and developmental success. In some animals, the neutral NH_3_ crosses the sperm membrane resulting in an alkaline interior that results in sperm motility. To test this, 100 μm of 100 mM NH_4_Cl was added to 1 ml of immobile sperm. Sperm motility was not induced at that concentration. On the other hand, sperm motility increases with time in sperm removed from the testis and diluted in pasteurized seawater. At zero minutes, sperm motility was less than 1%. At 20 min, sperm motility was less than 20%, and at 30 min, less than 40% and by 50 min sperm motility was high at about 80%. In a second experiment, motile sperm were added to oocytes that were either 3 h post-dissected (and rinsed) or freshly dissected (and rinsed). Fertilization was only observed in the freshly dissected oocytes. An increase in sperm motility with time, and a decrease of oocyte fertility with time, suggest that the sperm is first released into the seawater, where it may induce female spawning. This strategy is common to species, where the male-to-female proximity is distant in the wild. A third experiment was done to determine when germinal vesicle breakdown (GVBD) occurs in the absence of sperm. Twenty minutes post dissection (and rinse), 0/200 eggs showed GVBD; at 1 h 5 min, 5/200 oocytes showed GVBD; at 1 h 50 min, 5/200 showed GVBD; at 2 h 35 min, 8/200 showed GVBD; and at 3 h 35 min, 17/200 showed GVBD. In the presence of sperm, GVBD occurs almost immediately. These results indicate that sperm induces GVBD in *S. karankawa* oocytes.

An additional set of small experiments was done to determine the best seawater recipe for developing embryos, to determine if dechorionated embryos would develop normally, and to estimate the production of gut enzymes in early stage and late-stage tornaria. The first experiment consisted of rearing fertilized eggs in filtered and pasteurized seawater or in the artificial seawater Jamarin U (Jamarin La. Co., Osaka, Japan). Those in Jamarin U showed a higher fertilization success, and after 4 h, embryos in Jamarin U had developed more synchronously. A second set of experiments was done to dechorionate embryos and to determine if they would develop normally. Trypsin and protease did not weaken the chorion nor negatively affect development. Further embryos were passed through nitex meshes of 100 μm and 75 μm, but the chorion passed through intact, and development was normal. Dechorionation was achieved using a pair of fine-pointed tungsten needles at the 2-cell, 4-cell, 8-cell, and 16-cell stages. Development was normal, indicating that this species is suitable for experimental manipulations, including microsurgery, and cell injection investigations. No difference in chorion strength was apparent when dechorionated at newly fertilized versus 2-cell stage embryos. For the third experiment, 80-h embryos were incubated in an enzyme medium for esterase, and alkaline phosphatase and no staining was apparent. This shows that early tornaria either do not produce these digestive enzymes or the protocol was done too early before the enzymes are produced. Seventeen-day-old larvae showed a strong alkaline phosphatase reaction after incubation for 10 h, indicating cell differentiation and function of the gut. Dark purple staining showed in the gut, pillae of the ectoderm and the apical plate. At this same developmental time period, no esterase activity was detected.

#### Development

**Fig. 1:**
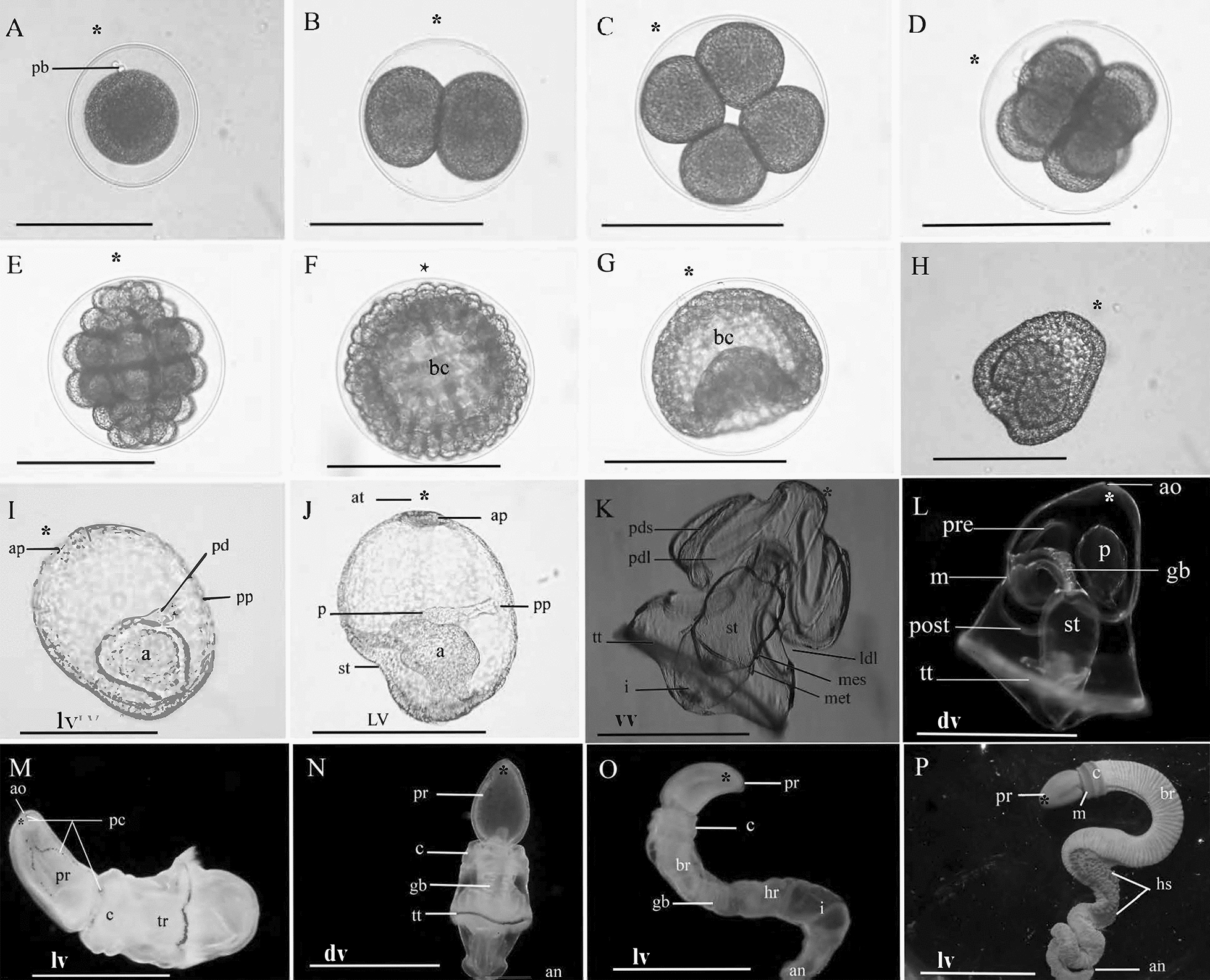
Development: light micrographs of cleavage and gastrulation in *Schizocardium karankawa.* **A** Zygote with polar bodies. **B** 2-cell stage. **C** 4-cell stage. **D** 8-cell stage. **E** 32 cell stage. **F** Blastula. **G** Mid-gastrula. **H** Hatching gastrula. **I**–**K** Early larval development in *Schizocardium karankawa*. **I** Lateral view of 23 hpf stage embryo at the time of mouth, stomach, and apical tuft formation. **J** Lateral view of developing tornaria larva at the time of protocoel formation. **K** Dorsal view of tornaria larva with mesocoel and metacoel, 30-day post-fertilization. **L** Late-stage larva with gills, 50-day post-fertilization. **M** Newly settled juvenile worm retained the larval pigment cells and the apical organ, feeding bands and telotroch. The larval apical organ develops into an outward projecting exploratory organ. **N** Juvenile worm showing the formation of the proboscis, collar, and trunk, 68-day post-fertilization. **O** Juvenile worm with the three regions of the trunk (branchial, hepatic, and intestine), 85-day post-fertilization. **P** Adult worm with hepatic sacs. *a* archenteron, *af* anal field, *an* anus, *ao* apical organ, *ap* apical plate, *at* apical tuft, *bc* blastocoel, *br* branchial region, *c* collar, *gb* gill bars, *hr* hepatic region, *hs* hepatic sac, *i* intestine, *ldl* lower dorsal lobe, *m* mouth, *met* metacoel, *mes* mesocoel, *p* protocoel, *pc* pigment cells, *pdl* primary dorsal lobe, *pds* primary dorsal saddle, *pre* preoral loop of the circumoral ciliary band, *post* postoral loop of the circumoral ciliary band, *pp* protocoel pore, *pr* proboscis, *tt* telotroch. Scale bars: **A**–**H** = 150 μm; **I**, **J** = 200 μm; **K**, **L** = 1150 μm; **M** = 3300 μm; **N** = 2100 μm; **O** = 2500 μm; **P** = 2 cm

Worms were collected starting on February 7, 2001, but gravid animals and successful fertilization were not achieved until February 20, marking the start of that reproductive season. The last fertilizations gotten, marking the end of the season, was April 16. These were not gravid: the oocyte nuclei were mostly central, and many were small, because yolk had not entered the vitelline envelope. The largest oocytes were 140 μm. On February 19, more worms were collected, and a small incision was made into the dorso-lateral gonads, releasing oocytes or sperm. Oocytes were 138 μm, some were slightly oblong, and only a few were very early oocytes with little vitelline space. Spermatid heads were 3.6 μm and tails were 40.0 μm, and very few were actively swimming. On Feb 20, mature sperm and oocytes were obtained. Oocytes were rinsed and fertilized with diluted sperm. The jelly layer was visualized with India ink at 300 μm in diameter. Within 4 min, the fertilization membrane expanded with the germinal vesicle still present. By 30 min post-fertilization, the nucleus migrated from a central location to a side of the oocyte. First and second polar bodies were observed 1.5 h post-fertilization (Fig. 1A). The embryo was 117 μm and the expanded envelope 159 μm. The first cleavage event began at 2 h and was completed at 2 h 30 min with oblong (66 μm by 90 μm) blastomeres (Fig. 1B). Nuclei were apparent at 2 h 20 min. Four cell stage was complete at 3 h 40 min (Fig. 1C), 8 cell at 3 h 55 min (Fig. 1D), 16 cell at 5 h with polar bodies atop (animal pole) four tiers of blastomeres including two central macromeres, vegetal and animal mesomeres which then divide to form two tiers of micromeres (Fig. 1E). A blastula (Fig. 1F) was formed at 6 h 15 min, and gastrulation commenced at 9 h. The gastrula soon took a hemispherical form (maximum 163 μm wide) with a large blastopore and no cilia (Fig. 1G). At 11 h 45 min post-fertilization, short cilia begin to rotate the embryo in the vitelline space at the speed of 35 s per revolution. Three tori-shaped tiers of cells then develop around the blastopore, reducing its size. Hatching occurred during the developmental period when the archenteron appeared as a dense mass (Fig. 1H).

At 22 h 30 min post-fertilization, the apical end of the early larva flattened and thickened (pre-apical tuft). There were no ciliated bands (Fig. 1I). At 34 h 30 min post-fertilization, the larva had a well-developed apical tuft included flagella with a length of 58 μm, an apical retractor muscle, buccal cavity oesophagus, stomach, intestine, and the site of the feeding and telotroch bands were apparent, but the cilia were uniformly 14 μm in length (Fig. 1J). Coeloms were absent. At 50 h post-fertilization, the apical plate showed some pigment and a few mesenchyme cells just below it. A well-formed pericardium pulsated once every 10 s and was in direct contact with the protocoel. Podocytes were apparent on the protocoel duct connected to an excretory pore (Fig. 1J). No mesocoels or metacoels were present. External cilia were 23 μm in length, uniform, with a predominantly downward beat. At 58 h post-fertilization larva had well-developed feeding bands, larval rotation was clockwise when viewed from the apical pole and the algae *Nanochloropsis* was ingested. At 72 h post-fertilization, a pair of dark eyespots were apparent. Table 1 shows the developmental time periods of *Schizocardium karankawa* from February 25 and April 10, 2001, at 19 °C and 23.5 °C, respectively.

**Fig. 2:**
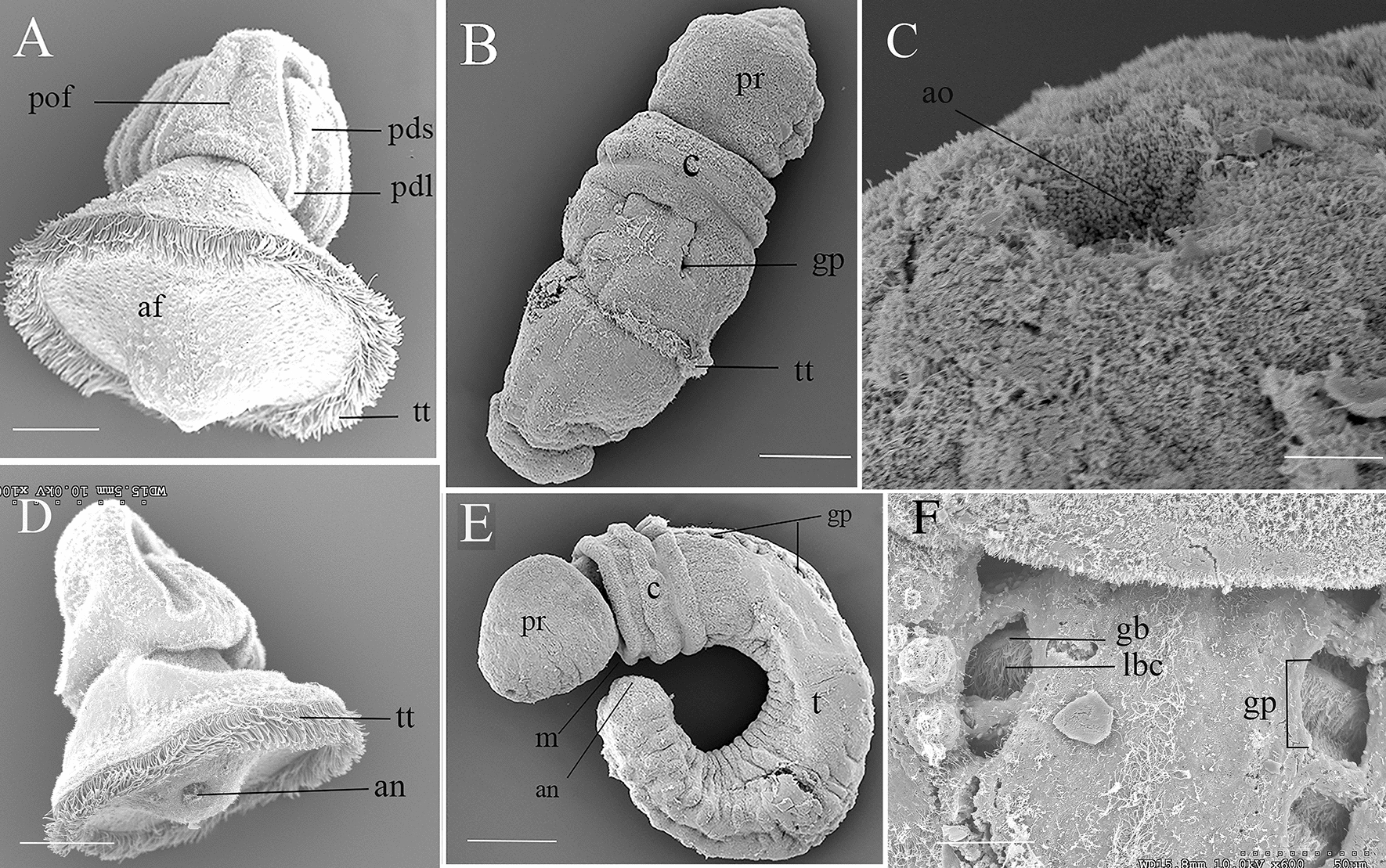
SEM images of *Schizocardium karankawa.* **A** Ventral view of a 1ate stage tornaria equivalent to Fig. 1K. **B** Dorsal view of a two gill pore stage juvenile worm. **C** Apical organ of the juvenile worm develops from that of the larva. In life, the organ is outward projecting and exploratory, but under SEM it is a concave fixation artifact. **D** Dorsal view of a late stage tornaria with ciliated anus. **E** Lateral view of a juvenile worm at the seven-gill pore stage. Elongation occurs primarily at the posterior trunk. **F** Details of the gills at the three-pore stage. The gill bars with lateral bar cilia can be seen through the gill pores. *af* anal field, *an* anus, *ao* apical organ, *c* collar, *gb* gill bars, *gp* gill pores, *lbc* lateral bar cilia, *pdl* primary dorsal lobe, *pds* primary dorsal saddle, *pof* postoral field, *pr* proboscis, *pt* protuberances, *t* trunk, *tt* telotroch. Scale bars: **A**, **D** and **E** = 500 $\mathrm{\mu m}$; **C**, **B** = 30 $\mathrm{\mu m}$, **F** = 50 $\mathrm{\mu m}$

More worms were collected, and fertilizations done between March 13 to April 12, 2013, and reared through tornaria (Fig. 1K) and metamorphosis to the juvenile worm stage (Fig. 1L to 1O). We do not know the start of the 2013 reproductive season, but the last worms collected on April 12 included 8 females and 9 males. Very few to no oocytes were found, marking the end of the 2013 season, 4 days earlier than the 2001 season. These embryos were maintained in culture through tornaria (Fig. 1K), late-tornaria (Figs. 1L and 2A), though metamorphosis (Figs. 1N and 2B), to the 6-gill pore stage (Fig. 1O). Metamorphosis was asynchronous, with the earliest sibling tornaria settled at 50-day post-fertilization (Fig. 1L). Others had not metamorphosed or settled 85-day post-fertilization, when our observations ended. A cavity in the anterior proboscis shows the site of the larval apical tuft (Fig. 2C). The tornaria anus was ciliated, and a vertical band of cilia connected the mouth to the telotroch, then the telotroch to the anus (Fig. 2A, D). Like other indirect developing acorn worms, metamorphosis was not catastrophic but a gradual process that incorporated larval tissue into the adults, through elongation and regional (proboscis, collar, trunk) elongation, with the telotroch maintained into the early juvenile stage (Fig. 2B). The cilia of the larval apical organ were lost in the worm, but the pigment cells were maintained. This most apical point appears to maintain a sensory function into the early juvenile worm stage (Fig. 1M). It projects forward and actively explores the surface and is positioned between the larval cup ocelli In SEM, its concave appearance is a fixation artifact (Fig. 2C).

Pigment granules that marked the larval feeding bands were maintained on the juvenile proboscis and anterior collar (Fig. 1M), whereas those of the telotroch were easily identifiable into the 8-gill slit stage (compare Fig. 1N, O). The pharyngeal gills developed in the late tornaria, where they did not obviously serve a function, because the ectodermal pores had not opened to the exterior until the juvenile worm stage. About 1-month post-metamorphosis at the 6 gill-pore stage, the (endodermal) gill bars could be seen though the (ectodermal) gill pores, and protuberances appear on the trunk (Figs. 1P and 2E). The gill bars at this stage have lateral cilia (Fig. 2F). Elongation by cell proliferation is most evident in the post-telotroch trunk.

**Fig. 3:**
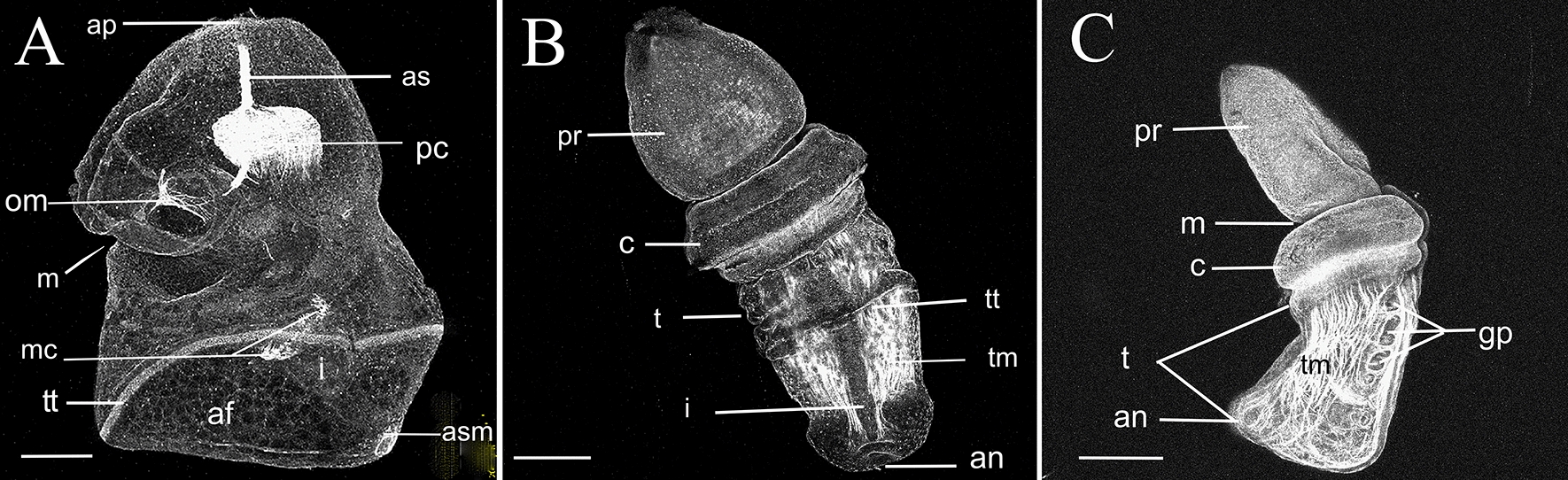
Confocal microscope images of a *Schizocardium karankawa* **A** tornaria larva with apical tuft, telotroch, and anal sphincter muscles. This larval stage is directly comparable to Fig. 1K. **B** Dorsal view of a juvenile worm with distinct proboscis, collar, and trunk. **C** Left lateral view of a 4-gill pore stage juvenile worm with long proboscis, mouth, collar, and trunk. *af* anal field, *ap* apical tuft, *an* anus, *as* apical strand, *asm* anal sphincter muscles, *c* collar, *gp* gill pore, *i* intestine, *m* mouth, *mc* muscles of the metacoels, *om* oesophogeal muscles, *pc* pericardial sac, *pr* proboscis, *t* trunk, *tm* trunk muscle, *tt* telotroch. Scale bars = 200 $\mathrm{\mu m}$

We performed confocal microscopy on actin (phalloidin) labeled, late-stage tornaria (Fig. 3A), and early and 4-gill pore stage juvenile worms (Fig. 3B, C). The 50-day post-fertilization stage tornaria had a well-developed apical retractor strand that connects the contractile pericardial sac to the apical tuft (Fig. 3A). In living animals, it frequently and rapidly contracted to form a temporary dimple in the animal pole. Another set of contractile muscles connected the pericardium with the oesophagus. The oesophagus was muscular dorsally (Fig. 3A). Together, these muscles were responsible for a ‘coughing’ behaviour that would clear the mouth of algae. At this stage, muscle cells began to develop in the early mesocoels, and an anal sphincter muscle was evident (Fig. 3A). Newly metamorphosed worms had limited circular muscle fibres in the proboscis. Little muscle was detected in the collar. The trunk has well-developed anterior to posterior muscle bands (Fig. 3B). These began to form more discrete, paired dorsal and ventral–lateral bundles around the 30-day post-settlement stage (Fig. 3C). The dorsal pair are more densely packed than the ventral–lateral muscles. However, the ventral ones are more extensive, as they are in adults. The paired buccal and perihaemal muscles of adult worms, which develop from anterior extensions of the trunk coeloms, are not apparent at this stage. Well-defined muscle cells form a sphincter around each of the gill pores (Fig. 3C).

#### External adult features

**Fig. 4:**
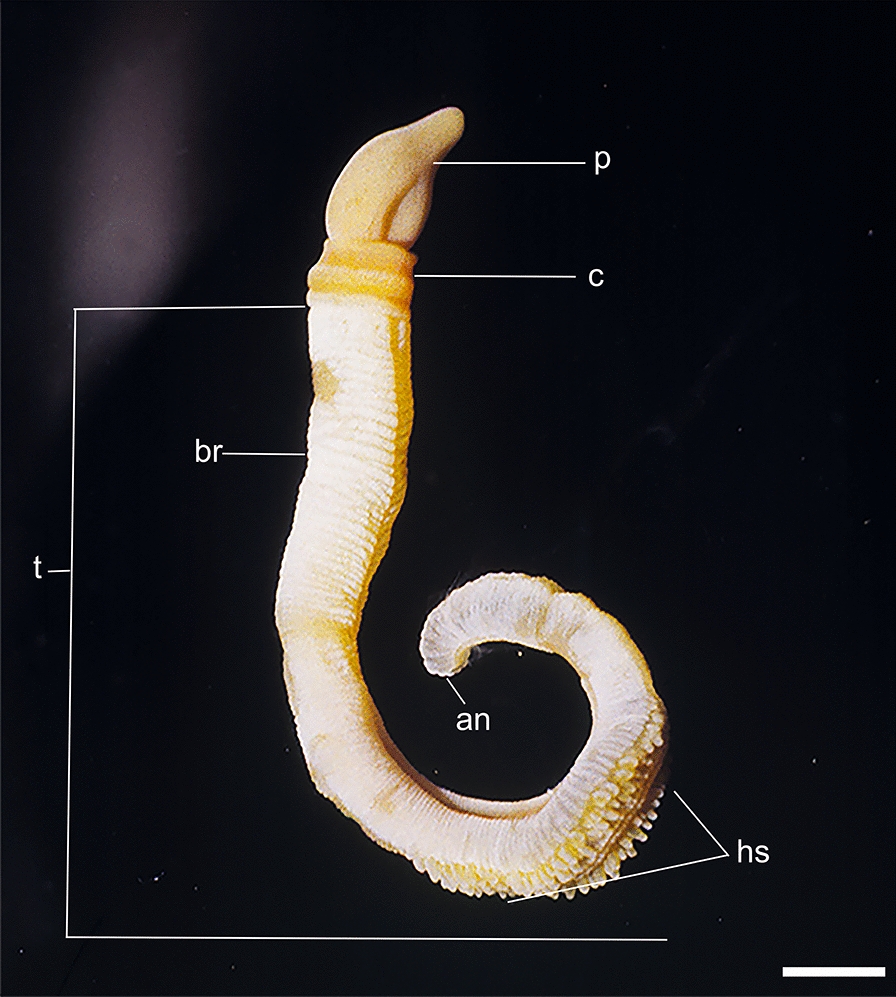
Photograph of an adult living specimen of *Schizocardium karankawa.* sp. nov, *an* anus, *br* branchial region of trunk, *c* collar, *hs* hepatic sacs, *pr* proboscis, *t* trunk, *vg* ventral groove. Scale bar = 1 cm

Total body length of the type specimen after fixation was 60.7 mm. The proboscis length was 5.1 mm by 4.6 mm wide. The collar was 2.3 mm long and 3.4 mm wide, and the trunk 53.3 mm long (branchial region 25.6 mm long, the hepatic region was 20.6 mm, and the caudal region was 7.1 mm), while the anterior width of the trunk was 3.4 mm, and posterior width was 2.1 mm. This is generally smaller than *Schizocardium peruvianum* and *Schizocardium californicum* [21]. The first paratype whole body length was 66 mm, proboscis length was 8 mm, and the width was 5 mm. The collar was 5 mm long and 3 mm wide. The first third of the hepatic region sacs had one pair, then two pairs of sacs, then mid-hepatic region the sacs were arranged in rows of three to four pairs (Fig. 4). The posterior third of the hepatic region had one or two pairs, the outer more well-developed than the inner which were small and oval, unique to this species. The synapticula that bridge the primary and secondary gill bars are medium developed. Gill pores were diminutive but numerous, estimated at 120–150 pairs. The gill bars were long, extending almost the depth of the pharynx, from dorsal to ventral, except for a ventral hypobranchial ridge, characteristic of the genus. The very small pores and large pharynx may be an adaptation to deposit feeding on very fine sediment. We found no evidence of a filter-feeding current through the pharynx.

#### Internal adult features

**Fig. 5:**
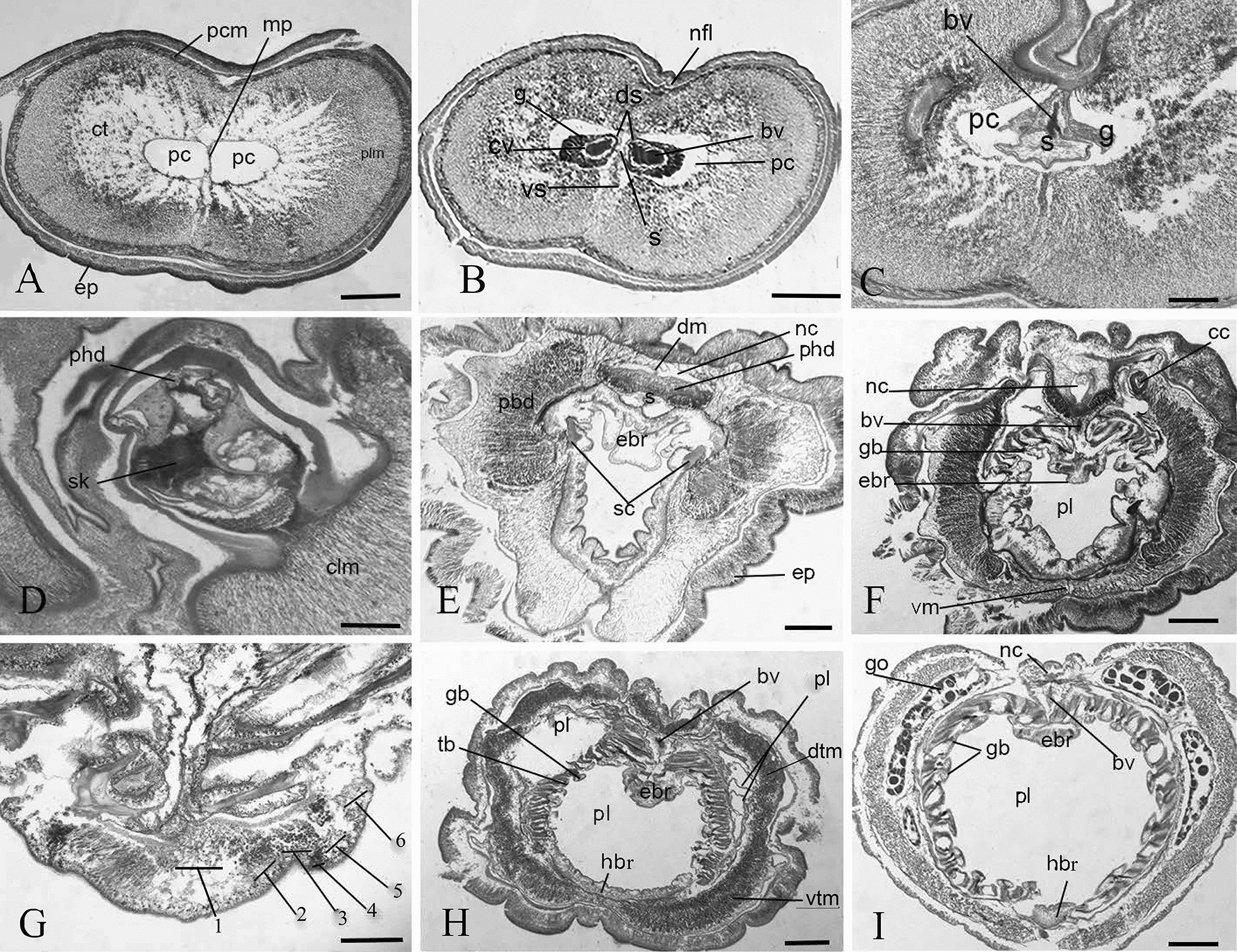
Light microscopy transverse histological sections of *Schizocardium karankawa* sp. nov. **A** Anterior end proboscis shows proboscis coelom filled with muscle fibers and the central portion of proboscis coelom divided via sagittal muscle plate into right and left. **B** Proboscis coelom with the paired tubular extensions of the cardiac vesicle, glomerulus, and stomochord or the heart–kidney–stomochord complex. **C** Posterior end of the heart–kidney–stomochord complex. **D** Neck enveloped by tissue of the anterior collar, showing the keel of the proboscis skeleton. **E** Collar region showing well-developed, paired peribuccal and perihaemal diverticula and the paired cornua of the proboscis skeleton. **F** Anterior trunk shows one of the collar canals, and the epibranchial ridge. **G** Six cell zones of the epibranchial ridge. **H** Pharyngeal region of the trunk with the primary and secondary (tongue) bars, and trunk muscles. **I** Pharyngeal region of the trunk with gonads. *bv* blood vesicles, *cc* collar canal, *ct* connective tissue, *cv* cardiac vesicles, *clm* collar longitudinal muscles, *dm* dorsal mesentery, *ds* dorsal septum, *dtm* dorsal trunk muscles, *ebr* epibranchial ridges, ep, epidermis, *g* glomerulus, *gb* gill bars, *go* gonads, *hbr* hypobranchial ridge, *nc* nerve cord, *nfl* nerve fiber layer, *pbd* peribuccal diverticula, *pc* proboscis coelom, *pcm* proboscis circular muscles, *phd* perihaemal diverticula, *pl* pharynx lumen, *ppd* peripharyngeal diverticula, *s* stomochord, *sk* skeletal keel, *sc* skeletal cornua, *vm* ventral mesentery, *vtm* ventral trunk muscles. Scale bars: **A** 450 μm; **B** = 1000 μm; **C**, **D** and **H** = 500 μm; **E** = 400 μm; **F**, **I** = 600 μm; **G** = 150 μm

The proboscis ciliated epidermis was 400–500 μm thick. The proboscis cavity was filled with diffusely arranged longitudinal muscle fibres surrounded by a layer of a circular muscle of almost the same thickness as the epidermis (Fig. 5A), with a well-developed nerve fiber layer between them. The proboscis coelom was filled with a connective tissue arranged in a small plate, and the center of the coelom was divided into distinct left and right portions via a thin muscle plate, each with a cardiac vesicle tube and glomerulus enveloping the anterior end of a bifurcated stomochord from which the genus gets its name (Fig. 5B). The right-side cardiac vesicles extended more anteriorly than the left. Each cardiac vesicle has a narrow coelom that separated it from the glomerulus that surrounds it. Each glomerulus has a ring shape anteriorly, and crescent shape with a thick ventral–lateral wall posteriorly (Fig. 5B). Posteriorly, the glomeruli and the cardiac vesicles connect in conjunction with the appearance of the stomochord. The vermiform process of the stomochord was short. A single proboscis pore was located on the left of the dorsal midline of the proboscis neck, like *Schizocardium peruvianum* and *S. californicum* [21]. At this point, a thick dorsal septum connected the stomochord with the dorsal wall of the proboscis, forming a deep dorsal groove in the proboscis (Fig. 5C). A limited ventral septum may attach to the ventral wall of proboscis but was broken in transverse sections (Fig. 5C). The stomochord radius was greatest and variable shaped due to intrusions of the surrounding basal lamina sheath, forming diamond, and star shapes, with a horizontal mesh of tissue in its central part. The stomochord lacked a lumen anteriorly, but posteriorly a small one appeared and disappeared sequentially. The stomochord lacked blind pouches. At this level of proboscis, the thickness of the epidermis and the circular muscle were about equal.

At the posterior proboscis and anterior collar, the ventral septum disappeared while the dorsal mesentery continued. The proboscis skeleton had a prominent keel and chondroid tissue (Fig. 5D). The skeletal keel was short, concave dorsally and convex ventrally (Fig. 5D). The dorsal collar mesentery continued and a ventral one was lacking. More posteriorly, in the anterior collar, the epidermis was enlarged (Fig. 5E). The skeleton bifurcated into paired cornua and a wide anterior collar neuropore was found. The collar nerve cord had no lumen like the other species of *Schizocardium* [21]. A dorsal mesentery extended from anterior to mid-collar with no evidence of the ventral one. More posteriorly, at the termini of the skeletal cornua, a ventral mesentery began in conjunction with the dorsal one. The buccal cavity of the posterior collar had a long and large epibranchial ridge that continued into the trunk pharynx lumen (Fig. 5E–I). There was a posterior collar neuropore. The pharynx epibranchial ridge had six zones of cell types, like the epibranchial ridge of *S. brasiliense* [26] (Fig. 5G). All zones were ciliated. Zone one, or the medial zone of cells, were lightly stained and transparent. The adjacent pair of cells, or zone two, were slightly biconcave nuclei present in a dark pink color with trichrome stain. The margin of zone three was biconvex shaped, resulting in a pair of grooves with dark stained cells with long cilia. The cells of zone four had a tapered base containing a large vesicle and distally positioned nuclei. The cells of zone five had large granular vesicles. Cells of zone six were comprised of columnar cells with apical nuclei. Under these zones was a thick epithelial nerve layer (Fig. 5G).

The branchial region of the trunk had left and right collar canals. The dorsal nerve cord of the trunk had small lacunae that appeared and disappeared anteriorly, but none posteriorly (Fig. 5H). The pharynx had a ventral mesentery. The boundary of the pharynx lumen was demarcated by primary and secondary (or tongue) gill bars. The secondary bars had peripharyngeal cavities (Fig. 5H, I). There was a well-developed dorsal blood vessel. Anteriorly, the pharyngeal trunk longitudinal muscles were thickest laterally and tapered dorsally and ventrally (Fig. 5F), whereas in the posterior pharynx region, the thickness was less variable (Fig. 5H, I). The ventral digestive pharynx narrowed from anterior to posterior until it was a greatly reduced hypobranchial strip. The posterior pharynx had well-developed dorsal and ventral blood vessels and dorsal lateral gonads (Fig. 5I). The posterior end of the trunk was damaged, but the intestine had a thick and winding wall, and a few gonads were found at the ventral side of the trunk. This region of the trunk had well-developed dorsal and ventral blood vessels.

#### Phylogeny

**Fig. 6:**
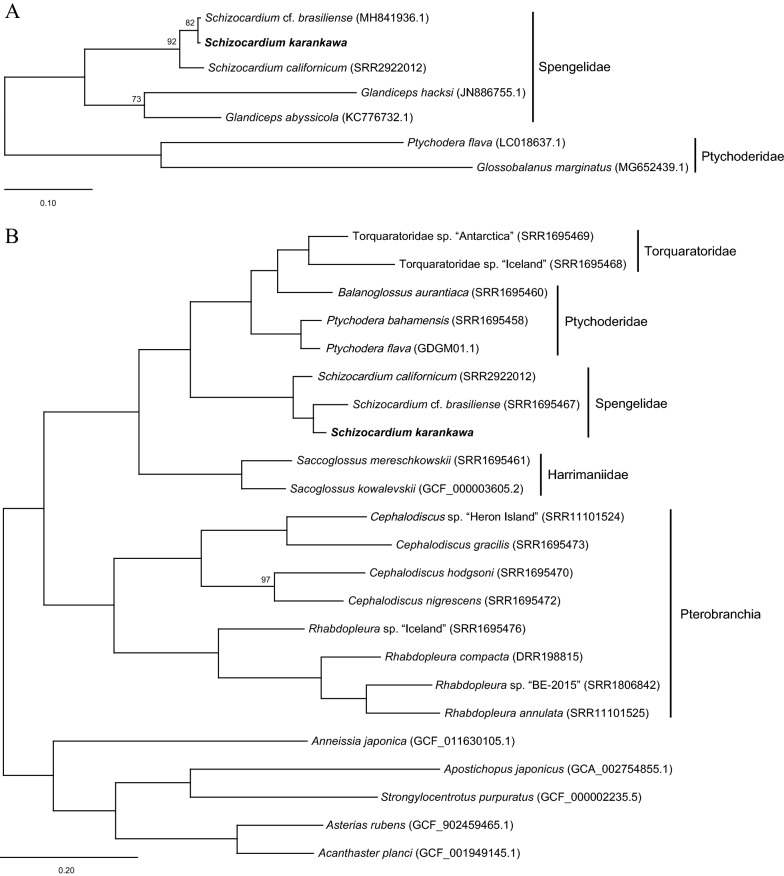
Phylogenetic analyses. **A** Maximum likelihood phylogeny of Spengelidae based on 16S showing near zero branch lengths separating *Schizocardium karankawa* and ‘*Schizocardium* cf. *brasiliense’* (sensu [22] and [68]) and the close relationship of *S. karankawa* and *S. californicum*. IQ-Tree 2 rapid bootstrap support values less than 100 are shown at each node. Ptychoderidae was used to root the tree. **B** Maximum likelihood phylogeny of Hemichordata based on 7948 genes totaling 1,504,053 amino acids positions. IQ-Tree 2 rapid bootstrap support values less than 100 are shown at each node. Echinodermata was used to root the tree

The full-length 16S sequence obtained from the Texas coast *Schizocardium karankawa* transcriptome differed from the 16S sequence obtained from the Mississippi coast *Schizocardium* cf. *brasiliens* mitochondrial genome (MH841936.1) at only 7/1362 shared positions (excluding a short stretch of 12 Ns in the *Schizocardium* cf. *brasiliens* sequence not considered in the analysis), resulting in an uncorrected p-distance of 0.005. For this reason and their proximity, we regard these two populations from the Gulf Coast of the USA as *Schizocardium karankawa*. For comparison, 16S of *Schizocardium karankawa* and *Schizocardium californicum* differed at 59/1365 shared positions, resulting in an uncorrected p-distance of 0.043. Phylogenetic analysis of 16S recovered *Schizocardium* monophyletic with *Schizocardium karankawa* sister to *Schizocardium* cf. *brasiliens*, albeit with moderate support (bs = 82) and *Schizocardium* sister to the other sampled genus of Spengelidae, *Glandiceps*, with maximal support (Fig. 6A).

Our phylogenomic pipeline retained 7,948 OrthoGroups totaling 1,504,053 amino acids in length with 58.5% missing data after concatenation. Phylogenetic analysis of this matrix yielded a topology consistent with the current understanding of hemichordate phylogeny (Fig. 6B). Except for Ptychoderidae, all hemichordate families were recovered monophyletic with maximal support. As observed in previous studies, Torquaratoridae was nested within Ptychoderidae. Spengelidae was recovered as the sister taxon of the Ptychoderidae–Torquaratoridae clade with maximal support. *Schizocardium karankawa* was recovered as sister to *Schizocardium* cf. *brasiliens* with maximal support. Here, we regard them as the same species, *S. karankawa*, based on the US Gulf coast population locations and the short phylogenetic distance between them. SI Table 1 includes the species and NCBI Accession numbers used in this study (Additional file 1: Table S1)

## Discussion

The genus *Schizocardium* includes *S. peruvianum* [46] from Peru, *S. braziliense* [46] from Brazil, *S. californicum* [21] from California, and *S. karankawa* sp. nov. from the Gulf Coast of the USA. A dichotomous key to the Spengelidae and a treatment of acorn worm zoogeography were provided in [21], topics that will not be treated here. Instead, our focus is the development of *S. karankawa* and its utility as a new model species in evolutionary developmental biology.

The discovery of *Schizocardium karankawa* and its reproductive season, conditions for fertilization, method of dechorionation, and development is significant, because it permits embryonic injections and manipulations and a direct comparison with the closely related *Schizocardium californicum* [21], an emerging evolutionary developmental model species [5, 6, 27]. The embryos and larvae of these phylogenetically close sister species are indistinguishable, which permits investigations into whether similar molecular-developmental processes, as might be expected, regulate their development. There have already been some surprises in this area with more distantly related species, including the comparisons of the direct developer *Saccoglossus kowalevskii*, to the tornaria of *Schizocardium californicum* [5]. The early juveniles of *S. kowalevskii* and *S. californicum* share genes expression patterns of transcription factors that are specific to the proboscis and collar regions, such as *fez, dlx, six3, rx, otx, lim1/5, pax6, irx, barH,* and *otp* [27] [5]. In addition, an extended set of transcription factors play a conserved role in patterning the larval body plan in hemichordate and sea urchin [47]. The larval apical region of sea urchin [48] and hemichordate [49] express *fezf.* Notable differences include the *Wnt* family of genes, including a component of 13 subfamilies that play a central role in embryonic recruitment to cell fate creation and patterning. While all *Wnt* subfamilies are present in *Saccoglossus kowalevskii* [50], the sea urchin *Strongylocentrotus purpuratus* lacks *Wnt2* and *Wnt11* [51]. The following detailed morphological comparison of *Schizocardium karankawa* with distantly related acorn worm species and echinoderms, and then with *S. californicum* highlights, where we might expect to find further differences in the molecular development of acorn worm species and echinoderms.

Perhaps the most significant difference of *S. karankawa* compared to other acorn worms, with respect to experiments in evolution and development, is that gametes are liberated from a short surgical cut into a gonad, fertilization is easily achieved in room-temperature bowls, and embryos are numerous, transparent, and develop normally following dechorionation. These traits are ideal for fate map and surgical embryology, gene expression studies, blastomere injections or dissociation for single-cell sequencing. The embryos of *Schizocardium karankawa, Ptychodera flava* and the direct developer *Saccoglossus kowalevskii* have holoblastic and radial cleavage, and the first three cleavage divisions are generally equal, resulting in animal and vegetal blastomeres (Fig. 1A). At the fourth cleavage (16-cell), *P. flava* and *S. kowalevskii* embryos differ in the relative size of the vegetal micromeres and the orientations process of the whole cleavage spindles responsible for formation of the animal mesomeres. In *S. kowalevskii,* the mesomeres generally do end up prone within a single plane, like those of *P. flava* and *Schizocardium karankawa.* Each of these species forms a ciliated blastula that gastrulates to form a mouth via deuterostomy. At this larval developmental period, the acorn worm larva is directly comparable to those of indirect developing echinoderms. These ‘dipleurula’ larvae are gelatinous with preoral and perioral ciliated bands that transport food to the mouth and into the oesophagus [52] (Fig. 1K, L). The left protocoelom is dominant and extends a ciliated duct, which is lined with podocytes to the exterior via a left dorsal lateral pore (Fig. 1 I, J, L). The coelomic perihaemal diverticula of the metacoels (Fig. 3B) in hemichordates are homologous to the echinoderm perihaemal coelom [57], and the metacoelomic extensions around the collar pharynx (peripharyngeal diverticula) of enteropneusts are homologous to the peripharyngeal coelom of the echinoderms [58]. The dipleurula larval nervous systems, including an apical plate and ciliated tuft, are almost indistinguishable. The notable difference of tornaria to echinoderm larvae is an apical plate retractor muscle (Figs. 1K, 3A) and a multiciliated locomotory telotroch that is retained into the juvenile worm stage (Fig. 1 K, N). The genes involved in these tornaria novelties are unknown. Direct developing acorn worms have the telotroch [4, 59], evidence that the acorn worm ancestor developed via a larva that was lost in harrimaniids like *Saccoglossus*.

The tornaria of *Schizocardium karankawa* and* Schizocardium californicum* are indistinguishable. It is later in development, at the adult stage, where the differences are most apparent. *S. karankawa* differs from *S. californicum* in that the proboscis coelom is completely divided into left and right via a sagittal muscle plate. Its stomochord differs from the other *Schizocardium* species in that the vermiform process is short, its lumen is almost non-existent, and it lacks a blind pouch. The primary and secondary gill bars of *S. karankawa* are connected by approximately 40 synapticula. *Schizocardium peruvianum* have 30 and *Schizocardium californicum* have 10–20 [21]. Cambrian fossil acorn worms lack synaptaculae, but *Schizocardium,* ptychoderids and cephalochordates have them, suggesting three instances of parallel evolution, if they share a common developmental basis [60–62]. The gill pores of *S. karankawa* are small but abundant at approximately 120–150 pairs of pores. *Schizocardium* species have two discrete rows of hepatic sacs [21] except for *S. karankawa,* which has one, two, and three rows of sacs depending on the anterior to posterior position along the digestive trunk. This may be related to diet, or gene drift, an experimental design to determine the contribution of which we have not explored. The epibranchial ridge is long and large compared to the other species. The hypobranchial ridge is less distinct than that of *Schizocardium peruvianum* [21], and may be a homologue to the invertebrate chordate endostyle [26], the progenitor of the vertebrate thyroid gland [63].

Here we compared the development of phylogenetically distant and phylogenetically close acorn worms. Studies of the first are common, the latter rare. Rarer are studies that explore the intraspecific variation of acorn worms [64, 65]. Intensive sampling of many individuals within a species are needed to assess the standing morphological variation and the amount and type of molecular intraspecific variation in developmental genes. The most comprehensive intraspecific study of acorn worms compares the many *Ptychodera flava* variants from the Indian Ocean [66]. The other quantifies gill pore number in sympatric populations of the acorn worms *Saccoglossus bromophenolosus*, *Protoglossus graveolens* and the cephalochordate *Branchiostoma floridae* [65]. *Saccoglossus bromophenolosus* is a deposit feeder, *Protoglossus graveolens* a facultative filter feeder/deposit feeder, and *Branchiostoma floridae* an obligate filter feeder. Fluctuating asymmetry, a measure of developmental noise, was highest in *S. bromophenolosus* suggesting that its gill development has experienced a relaxation with the abandonment of filter feeding. Intraspecific comparisons on the amount of variation in the structure of the heart–kidney coelomic process, collar nerve cord, the collagenous nuchal skeleton and gill bars, the ectodermal ossicles [67], and their developmental genes are needed, since these are key developmental characters used in systematics, in evolutionary developmental biology, because intraspecific variation is what evolution works on. An important challenge for evolutionary developmental biology is to form links from phylogenetically distant and large-scale differences to phylogenetically close and small-scale differences. This will help reveal the scale dependence of independence of evolution, and whether the origin of body plans and novelties can be attributed to accumulated micro-evolutionary changes over long periods of time, to standing variation (or unusual variants that are not normally present), or to meso-evolutionary changes over short periods of time.

## Supplementary Information

**Additional file 1**. SI Table 1. ACBI accession numbers of the 16S rRNA and transcriptome gene sequences used in the molecular analyses.

## Data Availability

Not applicable.
