# Supplementary material for: The embryology, metamorphosis, and muscle development of Schizocardium karankawa sp. nov. (Enteropneusta) from the Gulf of Mexico
Source: EvoDevo. 2023 Apr 19;14:6. doi: 10.1186/s13227-023-00212-0 (PMC10114407; doi:10.1186/s13227-023-00212-0)
Supplement: Supplementary file 1 — Additional file 1. SI Table 1. ACBI accession numbers of the 16S rRNA and transcriptome gene sequences used in the molecular analyses. [file 13227_2023_212_MOESM1_ESM.pdf]

**Phylum**	**Subclade**	**Species**	**Data Source**	**NCBI Accession #**	**Citation**
Echinodermata	Asteroidea	*Acanthaster planci*	NCBI Genome	GCF_001949145.1	Hall et al. (2017)
Echinodermata	Asteroidea	*Asterias rubens*	NCBI Genome	GCF_902459465.1	Hennebert et al. (2015)
Echinodermata	Crinoidea	*Anneissia japonica*	NCBI Genome	GCF_011630105.1	Li et al. (2020)
Echinodermata	Echinoidea	*Strongylocentrotus purpuratus*	NCBI Genome	GCF_000002235.1	Sodergren et al. (2006)
Echinodermata	Holothuroidea	*Apostichopus japonicus*	NCBI Genome	GCA_002754855.1	Li et al. (2020)
Hemichordata	Harimaniidae	*Saccoglossus mereschkowskii*	NCBI SRA	SRR1695461	Cannon et al. (2014)
Hemichordata	Harimaniidae	*Sacoglossus kowalevskii*	NCBI Genome	GCF_000003605.2	Simakov et al. (2015)
Hemichordata	Pterobranchia	*Cephalodiscus gracilis*	NCBI SRA	SRR1695473	Cannon et al. (2014)
Hemichordata	Pterobranchia	*Cephalodiscus hodgsoni*	NCBI SRA	SRR1695470	Cannon et al. (2014)
Hemichordata	Pterobranchia	*Cephalodiscus nigrescens*	NCBI SRA	SRR1695472	Cannon et al. (2014)
Hemichordata	Pterobranchia	*Cephalodiscus* sp. “Heron Island”	NCBI SRA	SRR11101524	Li et al. (2018)
Hemichordata	Pterobranchia	*Rhabdopleura annulata*	NCBI SRA	SRR11101525	Li et al. (2018)
Hemichordata	Pterobranchia	*Rhabdopleura compacta*	NCBI SRA	DRR198815	?
Hemichordata	Pterobranchia	*Rhabdopleura* sp. “BE-2015”	NCBI SRA	SRR1806842	?
Hemichordata	Pterobranchia	*Rhabdopleura* sp. “Iceland”	NCBI SRA	SRR1695476	Cannon et al. (2014)
Hemichordata	Ptychoderidae	*Balanoglossus aurantiaca*	NCBI SRA	SRR1695460	Cannon et al. (2014)
Hemichordata	Ptychoderidae	*Ptychodera bahamensis*	NCBI SRA	SRR1695458	Cannon et al. (2014)
Hemichordata	Ptychoderidae	*Ptychodera flava*	NCBI TSA	GDGM01.1	Luttrell et al. (2016)
Hemichordata	Spengelidae	*Schizocardium californicum*	NCBI SRA	SRR2922012	?
Hemichordata	Spengelidae	*Schizocardium cf brasiliense*	NCBI SRA	SRR1695467	Cannon et al. (2014)
Hemichordata	Spengelidae	*Schizocardium karankawa*	This study	OP596160.1	This study
Hemichordata	Torquaratoridae	Torquaratoridae sp. "Iceland"	NCBI SRA	SRR1695468	Cannon et al. (2014)
Hemichordata	Torquaratoridae	Torquaratoridae sp. "Antarctica"	NCBI SRA	SRR1695469	Cannon et al. (2014)
